# Primary Aortoenteric Fistula after Nissen Fundoplication

**DOI:** 10.7759/cureus.2386

**Published:** 2018-03-28

**Authors:** Catherine Bixby, Pathanjali Sharma, Faisal Aziz

**Affiliations:** 1 School of Medicine, UNECOM; 2 Vascular Surgery, Penn State Hershey Medical Center, Hershey, USA

**Keywords:** aortoenteric fistula, hematemesis, nissen fundoplication

## Abstract

We present the rare case of a primary gastro-aortic fistula involving the native aorta and proximal stomach in a patient with a chronic gastric ulcer and prior history of Nissen fundoplication. Our case highlights the importance of keeping this rare and fatal condition as a differential diagnosis in patients with prior history of Nissen fundoplication surgery.

## Introduction

An aortoenteric fistula (AEF) is defined as an abnormal communication between the aorta and gastrointestinal tract. It can be fatal due to massive hemorrhage into the gastrointestinal tract. It is a rare condition, with a reported incidence of 0.04–0.07% [1]. AEFs are broadly classified into primary and secondary types based on the etiology. Most AEFs are secondary in nature, and occur as a complication of prior open abdominal aortic aneurysm repair [1]. In these cases, the fistula is between the aortic graft and the gastrointestinal tract. In the majority of these cases, the third or fourth part of duodenum is involved. Primary AEFs are more uncommon and refer to a communication between native aorta and gastrointestinal tract. We describe the case of an AEF, which is unique due to its primary nature and anatomic location. The purpose of this case report is to emphasize that clinicians should keep AEF in the list of differential diagnosis in patients who present with massive gastrointestinal bleeding. In patients with AEF, misdiagnosis and delayed intervention are associated with an overall mortality of nearly 100% [2].

## Case presentation

A 57-year-old male was brought to the emergency room after being found unresponsive following an episode of massive hematemesis. The patient had undergone cardiopulmonary resuscitation prior to arrival in the hospital. His past medical history was significant for severe pulmonary fibrosis, chronic back pain and gastrointestinal reflux disease (GERD). His past surgical history was significant for open Nissen fundoplication five years prior. Based on clinical history and physical examination, the diagnosis of hemorrhagic shock due to upper gastrointestinal bleeding was made. An emergent esophagogastroduodenoscopy (EGD) identified a large amount of blood clot and a bleeding ulcer along the lesser curvature of the stomach. Endoscopic thermal coagulation was used to treat active bleeding from the ulcer. Over the course of the next several hours, his condition continued to deteriorate and a chest radiograph now showed new findings of pneumoperitoneum. An emergent exploratory laparotomy was performed which revealed a large amount of hemoperitoneum in addition to a gastric perforation along the proximal greater curvature. In addition, an area of active bleeding was seen in the region of the posterior gastric fundus and a 1–1.5 cm gastric ulcer incorporated in the posterior portion of the patient’s fundoplication wrap was found. Palpation around the ulcer revealed a large amount of thrombus, which upon removal resulted in massive hemorrhage. Manual compression revealed a fistulous tract running between the gastric fundus and abdominal aorta, which had developed from a penetrating chronic ulcer posterior to the fundoplication wrap. Control of the supraceliac aorta was difficult due to significant inflammatory changes, adhesions, and scar tissue. To facilitate proximal control, a left anterolateral thoracotomy was done and the distal descending thoracic aorta was clamped. Following incision of the diaphragm, the fistula was isolated and stomach separated from the aorta. The distal esophagus directly above the gastroesophageal (GE) junction was stapled off, and the devitalized proximal stomach removed. An attempt was made to repair the involved aortic segment using pledgeted prolene sutures. Due to the friability of the native aorta, a definitive repair was performed by replacing this aortic segment with 20 mm diameter Dacron graft.

Post-operatively, the patient remained in critical condition and suffered from acute renal failure, necessitating continuous renal replacement therapy (CRRT). He went on to develop abdominal compartment syndrome requiring a second emergent laparotomy. On re-exploration, the remaining portion of the patient’s stomach was noted to have large ischemic patches extending into the pylorus, cecum, transverse and descending colon. Additionally, there were ischemic patches throughout the liver. A complete gastrectomy and subtotal colectomy were done. Despite multiple operations, his overall condition continued to deteriorate. Multiple conversations took place with the patient’s family who ultimately decided to withdraw care, given the poor overall prognosis and need for additional gastrointestinal reconstructions and lung transplantation.

## Discussion

The majority of AEFs are secondary in nature, occurring as complications of open aortic repair. This is most likely due to disruption of retroperitoneal tissues, open suture lines, and prosthetic graft infection [1]. Secondary AEFs can also form following endovascular aneurysm repair (EVAR) and are due to persistent endoleak with gradual increase in aortic size, graft migration, graft erosion or adjacent organ injury from metallic stents or fixation devices. Primary AEFs are much less prevalent with an incidence ranging from 0.04% to 0.08%. They are commonly attributed to aortic aneurysms, radiation therapy, tuberculosis, collagen vascular disease and foreign body ingestion [2]. AEF is also an uncommon complication of esophagectomy and gastric tubes [3]. In these cases, fistula formation is closely linked to anastomotic leak. Surgical procedures involving the gastroesophageal region are associated with an increased prevalence of local ischemic complications due to the segmental blood supply of the esophagus as well as its dependence on collateral vessels traveling long distances. Since the introduction of proton pump inhibitors, the indications for operative intervention for patients with GERD are now limited to only those patients who fail to respond to maximal medical management or develop severe complications from chronic reflux. Nationwide, the number of operations performed for GERD has dramatically dropped over the past two decades.

This case is unique for several reasons: this patient had no history of aortic pathology and the origin of the fistula was stomach rather than duodenum. To the best of the authors’ knowledge, there are only seven cases of gastro-aortic fistulae reported in the literature [4,5]. The most significant factors promoting gastric ulceration into the abdominal aorta following Nissen fundoplication are local ischemia from ligation of the short gastric vessels, local suture irritation and direct surgical trauma [6]. Ongoing post-surgical inflammation results in adhesions surrounding the fundal segment of the fundoplication wrap. Differential diagnosis of hematemesis is rather extensive and usually does not include aortoenteric fistula in absence of previous aortic operations. The findings of this case report suggest that diagnosis of AEF should be included in patients presenting with hematemesis and a history of prior gastric surgery. In cases where no definitive bleeding source from the gastrointestinal tract can be found, the initial, self-limiting bleeding could be a “herald bleed” [2]. This episode is usually followed by massive hematemesis and hemorrhagic shock. The interval between these two episodes is variable and can be as short as six hours and as long as eight months [4]. Diagnostic imaging modalities for AEF have high false negative rates. Computerized tomography (CT) is considered the most sensitive (50%) and specific (100%) diagnostic imaging modality [7]. Findings of CT scan which may suggest the presence of an AEF include the presence of air outside the gastrointestinal tract, periaortic fluid, aortic wall disruption or loss of fat planes between the bowel and aorta [7]. Evaluation with endoscopy is superior to barium contrast as it enables direct visualization of the bleeding site, adherent clot, or regions of ulceration and necrosis [1]. Limitations of endoscopy include its low sensitivity (20%) mostly due to inability to visualize the exact source in cases with massive bleeding [7]. AEF involving a fundoplication wrap is especially difficult to visualize. In these cases, computerized tomography angiography (CTA) can be a valuable diagnostic tool as it enables visualization of contrast extravasation into the bowel.

To summarize, AEF is a fatal diagnosis and non-operative management is associated with 100% mortality. Open surgical repair is the gold standard operation for treatment of AEF. Surgical options include extra-anatomic reconstruction with aortic ligation and in situ reconstruction with either cryopreserved aorta or rifampin soaked Dacron graft. Open operations are associated with high incidence of perioperative complications, including anastomotic rupture, acute kidney injury (AKI), pulmonary failure, wound dehiscence, graft stenosis, recurrent infection, and death. Benefits of an endovascular approach include lower morbidity and mortality compared to open surgical repair (14% vs 36%), reduced post-operative recovery time, and avoidance of aortic cross-clamping [8]. It should be emphasized that the lower morbidity and mortality of endovascular repair has been linked to only the first two post-operative years [9]. Adverse effects from endovascular repair include increased incidence of re-infection and recurrent bleeding.

## Conclusions

It is crucial to maintain a high index of clinical suspicion when confronted with a patient presenting with hematemesis or symptoms resembling a “herald bleed” especially in patients with the history of Nissen fundoplication. Early diagnosis and treatment are crucial in the treatment algorithm for such patients. A delay in diagnosis or missed diagnosis increases mortality risk for such patients.
